# Resilient infrastructure

**DOI:** 10.1038/s44172-022-00032-5

**Published:** 2022-11-03

**Authors:** Carmine Galasso, Janise McNair, Manabu Fujii, Zhijie (Sasha) Dong

**Affiliations:** 1 Department of Civil, Environmental & Geomatic Engineering, University College London (UCL), London, UK; 2 Department of Electrical and Computer Engineering, University of Florida, Gainsville, FL USA; 3 Department of Civil and Environmental Engineering, Tokyo Institute of Technology, Tokyo, Japan; 4 Department of Construction Management, University of Houston, Houston, TX USA

## Abstract

The climate crisis, the Covid pandemic and rapid urban growth amongst other factors are leaving populations increasingly vulnerable to breakdown in key infrastructure systems. This includes the built structures that form our living and working environment, the transport and communications networks that serve us and the utilities that enable us to live comfortably.

In this Viewpoint, four of our editorial board members outline some of the key challenges in maintaining infrastructure resilience in the face of crisis, along with urgent research needs and technology opportunities. Emerging themes included the vulnerability across all sectors of the poorest communities and the importance of integration and systems thinking to achieve true resilience.

**Figure:**
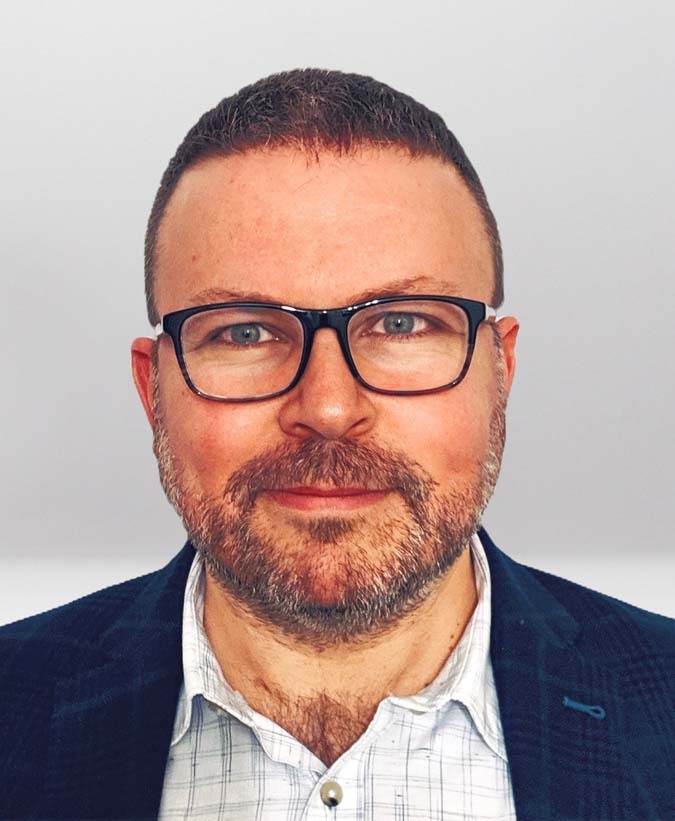
Carmine Galasso—Built Environment. Courtesy of Carmine Galasso

The global urban population is growing rapidly, with cities expanding or densifying. As a result, new assets are built on previously undeveloped land, and multiple infrastructure systems need to be expanded or improved to deliver more capacity. These changes fundamentally alter the exposure and vulnerability of people, their built environment, and their livelihoods to disaster risk. Most of the recent efforts to understand, model, quantify, and thus increase the resilience of cities, and key infrastructure considers static, present-day representations of built-natural-social environments despite their dynamic nature.

Furthermore, the root causes of multi-hazard and systemic risks often stem from human choices. Today’s decisions can either promote resilience building or amplify vulnerability and exposure. There is an urgent need to link urban changes to risk and resilience quantification to better represent the impact of today’s decisions in urban planning and policies on disaster risk and resilience projections.

Conventionally, the effect of natural hazards and subsequent demands imposed on engineered systems are investigated using computational/analytical models representing the system’s behavior in isolation. However, the actual performance and functionality of any urban system during and after a natural-hazard event also depend on the physical and functional performance of the surrounding systems. For example, suppose a building withstands an earthquake but is surrounded by collapsed buildings and no functioning water supply and electricity systems in the region. In that case, the building will suffer significant downtime despite its individual structural performance being acceptable. Assessing the actual performance of built urban infrastructures should be conducted on a regional scale, explicitly considering and modeling the complex interconnected response and recovery of various engineered systems. Examples of multi-disciplinary projects for assessing seismic risk and resilience at a regional scale are the SHAKEOUT^1^ and HAYWIRED^2^ projects conducted for prospective scenario earthquakes in California, in which the implied loss to different types of structures and distributed infrastructure and systems were assessed. Public response to the event, economic and societal impacts, recovery from the disaster, and preparedness for future events was also addressed.

Current approaches also generally focus on a single hazard, like an earthquake or a landslide, ignoring interactions between them (if any). However, hazardous events may occur simultaneously, cascadingly, or cumulatively over time, with potential interrelated impacts. For example, vulnerable and marginalized communities, such as the urban poor, can have their livelihood regularly reset by smaller floods or landslides, often resulting from poor planning decisions outside their communities. It is essential to look at multi-hazard scenarios covering a wide range of scales, from single high-magnitude events to repeated small disruptions, connecting intensive risk to extensive or even everyday multiple hazards and bridging the near-real-time priorities of the urban poor with longer-term strategic resilience planning.

Indeed, both the Sustainable Development Goals^3^ and the Sendai Framework^4^ call for greater protection of people disproportionately affected by natural hazards, such as the urban poor. When a bridge collapses, for example, the repair or replacement cost is only the tip of the iceberg. Loss of connectivity disrupts education, work, and community, degrading livelihoods and economic activity, particularly for the urban poor, that are asset poor and lack coping capacities. A pro-poor understanding of risk and resilience must include these lived experiences, especially the well-being of marginalized communities. The poor are the least resilient to disaster impacts and yet are under-represented in asset loss calculations. There is thus also an urgent need to redefine disaster risk and resilience quantification to go beyond the direct cost of physical damage and include impacts on the well-being and economics of those groups who suffer the most.

**Figure:**
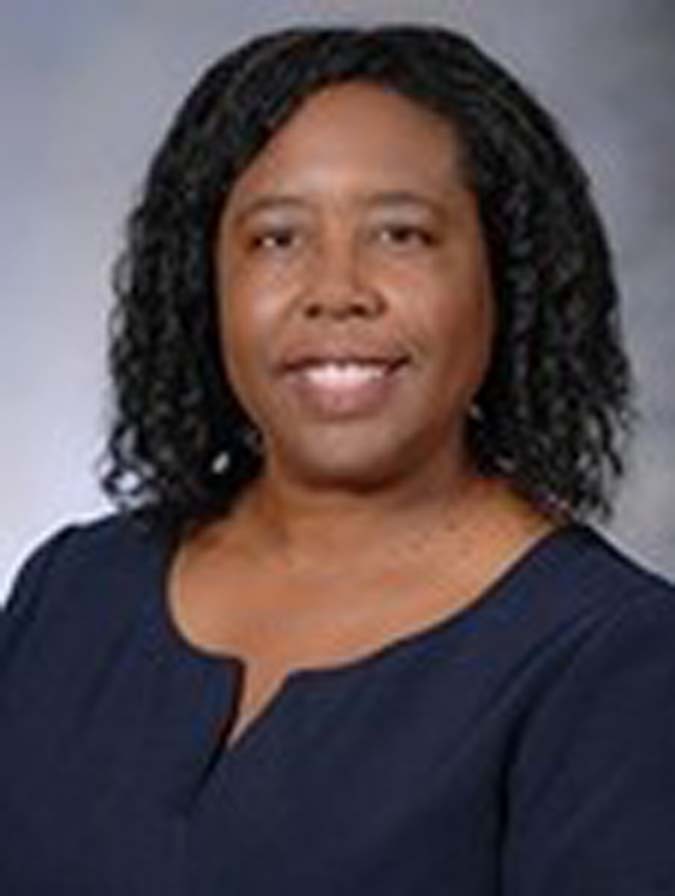
Janise McNair—Communications Networks. Courtesy of Janise McNair

The global economic, social, scientific, and entertainment infrastructure has become dependent on the ready availability of communication and network services, especially the availability of wireless services. This dependence has created opportunities for advancement in many areas of business, education, and social services. But it also reveals a crucial need for resilience in the communications infrastructure with respect to societal access, physical resilience, and security management.

Resilience in communication networks has a vital impact on society, and access to this infrastructure has become a key indicator of the ability of a community, country, or society to sustain operations in the presence of external dangers. The pandemic caused many industries to transition to an online format to survive. Former in-person services, from ordering food to purchasing goods to teaching and learning and corporate business environments, were moved to the online communications and networking space. This transition created opportunities for improved application delivery (Zoom, Google Meet, Cisco Webex, Apple Face Talk, Facebook Live, etc.) and increased bandwidth delivery to support the new applications. Without these resources, a more significant breakdown in business and services would have been evident. The necessity of an online infrastructure also revealed the dire consequences of insufficient communication resources at home, due to network access limitations, income limitations, and/or location limitations, where rural and remote communities, as well as low-income communities, did not have sufficient 5 G or Internet services to allow full participation in the online environment.

As with other physical infrastructures, another concern for communications access is the increasing number of disaster events. In 2019, the USA experienced 14 different billion-dollar disaster events—its fifth consecutive year to do so^5^. Such events may wipe out existing 5 G infrastructure, cutting off crucial communications and impeding the delivery of emergency services. A multi-tiered, multi-access approach is required that includes a combination of interoperable terrestrial, high-altitude, and satellite networks to maintain this crucial service even under dire conditions.

Beyond the physical communications structure, researchers are creating cyber-enhanced structures that inform the behavior of a physical system using cyber-based modeling, machine learning, and control. This includes (1) digital twin systems, a high-fidelity digital representation of a physical system that evolves with the physical entity through its life cycle, and (2) cyber-physical systems, which integrate computational and physical components to implement a cyber-controlled sensing and feedback process on a physical entity. This type of infrastructure is growing in several key industries, from agriculture to electric utility services. Next-generation power systems are evolving to the smart grid where the physical processes will achieve greater stability, efficiency, and robustness through the integration of cyber-based control, communication, and computation. However, these new capabilities introduce a new vulnerability: cyber-threats. The first confirmed blackout from a cyber-attack on a power grid happened in 2016 in Ukraine and caused a power outage that affected 225,000 customers^6^.

Potential disruptions to communications networks are increasing, from catastrophic incidents, including natural disasters or malicious security attacks, to system operation shortcomings, such as accidental security breaches and insufficient access to resources. The key to communications infrastructure resilience will be a focus on intentional and widespread societal access to communications services; creating heterogeneous and redundant (backup and alternative) systems for access methods that allow for rapid recovery of physical communications infrastructures; and continuing research on cyber-physical systems security management.

**Figure:**
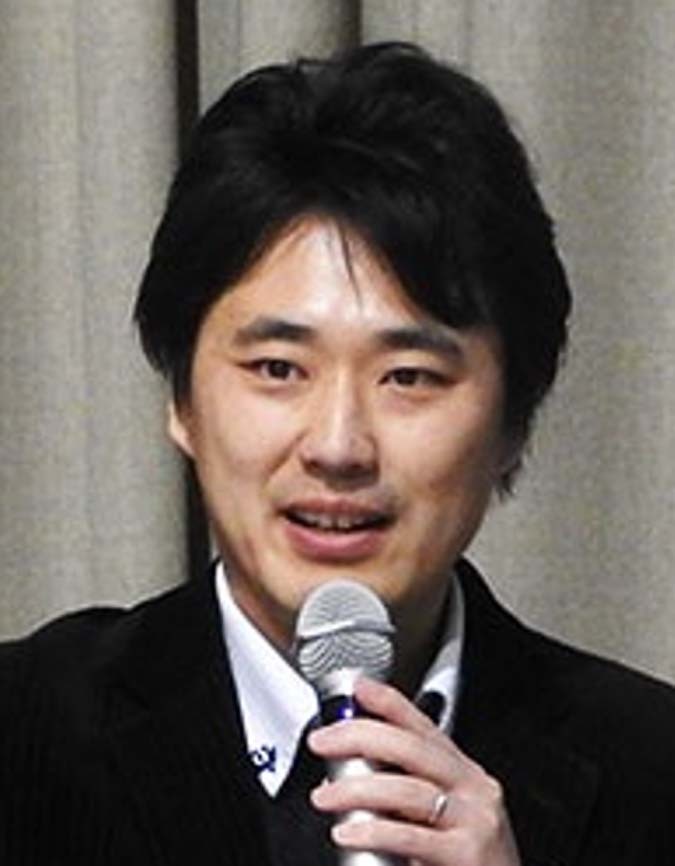
Manabu Fuji—Water systems. Courtesy of Manabu Fujii

Water infrastructure must be resilient to population and economic growth, extreme weather events, and the emergence of novel pollutants. As water demand increases, unconventional water sources from desalination, wastewater reclamation, and dehumidification are becoming a focus. Meanwhile, opportunities to integrate the management of water supply, sewage treatment, stormwater drainage, brine and sludge disposal, energy and resource harvesting, and environmental protection are also of great interest.

Initiatives to enhance resilience against water crisis have been undertaken for decades, particularly in water-stressed regions. In Israel, for example, the coastal desalination plant network (encompassing the National Water Carrier) has been active since 2005, supplying domestic water throughout the country with sewage being reused for agricultural purposes^7^. Singapore has expanded its reservoirs and has increased its domestic water reliance on desalination and NEWater (high-grade reclaimed water produced via microfiltration, reverse osmosis, and UV disinfection of secondary treated wastewater), with plans to reach more than 80% by the mid-22nd century^8^.

More recent initiatives integrate energy and environmental considerations to make the water infrastructure more resilient. Desalination technologies may combine reverse osmosis-based processes with emerging techniques utilizing sustainable energy such as solar water domes, wave-powered desalination, and modified solar stills^9^. Major opportunities in wastewater treatment include upgrading toward self-sustaining technologies via energy saving and recovery whilst efficiently removing pollutants. For example, anaerobic membrane bioreactors and annamox processes remove organic matter and nutrients without energy-consuming aeration. And anaerobic digestion and microbial fuel cell processes generate methane and electricity, respectively, concurrently with wastewater treatment.

Emerging chemical contaminants such as pharmaceuticals and personal care products, perfluoroalkyl and polyfluoroalkyl substances, microplastics and their transformation products, as well as disinfection by-products, are also of major concern. Reinforcement of real-time monitoring, and regulation, as well as identifying new treatment routes, and upgrading treatment efficiency, will avert major health and environmental crises^10^. Meanwhile, surveillance technology based on the measurement of genes and chemicals in sewage (namely wastewater-based epidemiology) is being put to practical use, for example, in early warning of the COVID-19 pandemic, leading to increased resilience of society against pandemics^11^.

Global sustainable and resilient water supplies will require both government and private-sector investment, particularly in the Global South. Decentralized off-grid technologies such as point-of-use treatment are considered an economically feasible short-term solution alongside centralized water distribution networks. For example, low-cost point-of-use treatment technologies have been developed for drinking water, including flocculation using biodegradable materials, biosand filtration, and disinfection using sunlight and nanomaterials; treatment products packed into sachets make simple technologies for use in developing communities^12^. Given that resilient water infrastructure is interconnected with many other sectors, its installation while minimizing trade-offs with energy and the environment will synergistically contribute to many of the Sustainable Development Goals related to poverty, hunger, and universal good health, strengthening resilience and the sustainability of society itself.

**Figure:**
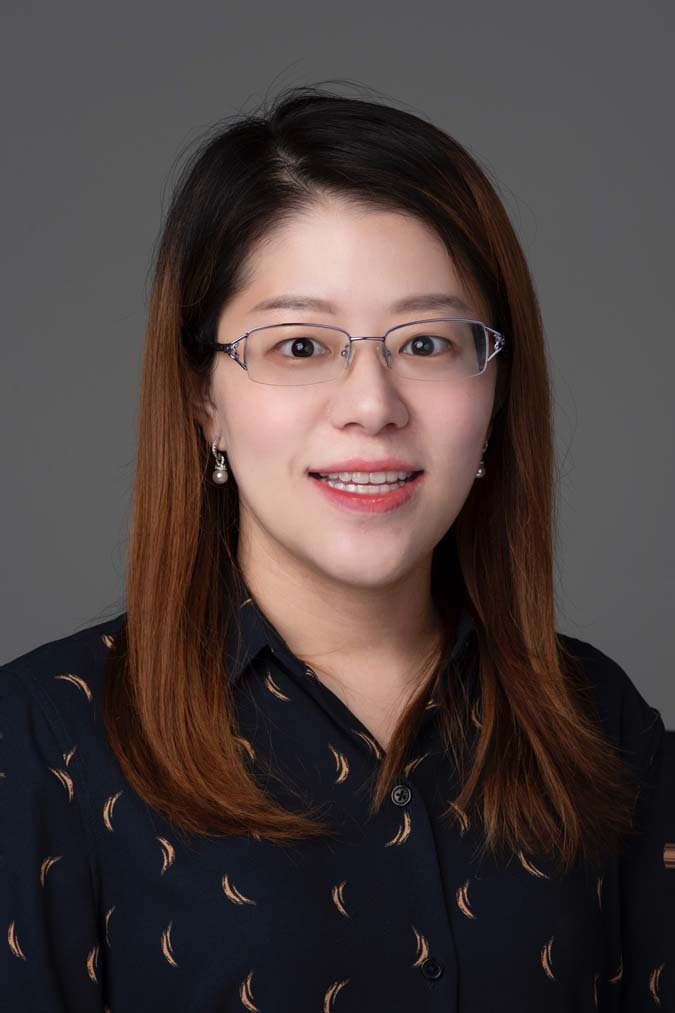
Zhijie (Sasha) Dong—Supply chains and transportation. Courtesy of Texas State University

In recent years, supply chain disruptions have received increasing attention due to the impact of unpredictable and uncontrollable events. For example, as Covid-19 has spread across the world and infected a huge population, major transportation activities halted due to a worldwide lockdown, which led to substantial interruptions in actual goods flows, and product and human mobility, and affected the entire supply chain. Taking the automobile supply chain as an example, China’s shutdown at the beginning of February 2020, resulted in nearly 50% of the global automobile manufacturers suffering a shortage of parts and components. Some production lines completely ground to a halt^13^.

Supply chain disruptions thus create shortages leading to higher prices and not only for high-end consumer products. Disruptions can also limit the availability of basic commodities such as generic drugs, energy, and food. If provisions are poorly distributed, it can be more difficult to identify their location, and individuals may have to travel further to get hold of them. This, along with higher prices due to lower availability^14^, means that the impact of supply chain disruptions on vulnerable populations is particularly dire^15^.

It is imperative to improve the resilience of the transportation network against all sources of external disruption to ensure the responsiveness and efficiency of supply chains and avoid a humanitarian crisis. We need to be able to rapidly identify alternative solutions to move goods from place to place effectively. Making optimal use of all the transportation networks (rail, air, water, road) can minimize the amount of freight and number of people that travel by road, reduce travel times, increase consumer and producer mobility options, and provide access to basic services for many vulnerable portions of the population. Seamless and collaborative multimodal transport management systems that maximize coordination and connection between the different transportation sectors can improve transport flexibility and strengthen supply chain resilience. One recent example of such a multimodal transport system is the On-Demand Multimodal Transit System, which combines a network of high-frequency trains and buses with on-demand shuttles to serve the first and last miles during a pandemic^16^. But such management systems are few and far between.

Current challenges facing the development and implementation of multimodal transport management systems arise from the need for interaction among different transport modes and various actors to ensure accurate, timely, and efficient communication. Emerging technologies, such as cloud computing, AR/VR technologies, social networking, wireless communication, and the Internet of Things enable new perspectives on the interaction and connectivity between different players. For example, as computing power increases exponentially and smart wireless devices get smaller, more affordable, and more capable, people, as well as devices, can be connected anywhere at any time. Such ubiquitous connectivity and network service could enable real-time and extended visibility of possible supply chains, efficient data exchange, and better flexibility to react to unexpected changes and disruptions across multimodal transport chains. Research focusing on the development of technology-driven solutions to integrate transportation networks will be a powerful tool for the development of more integrated and reliable supply chains.
