# Author Correction: Resilient Infrastructure

**DOI:** 10.1038/s44172-022-00036-1

**Published:** 2022-11-18

**Authors:** Carmine Galasso, Janise McNair, Manabu Fujii, Zhijie (Sasha) Dong

**Affiliations:** 1 Department of Civil, Environmental & Geomatic Engineering, University College London (UCL), London, UK; 2 Department of Electrical and Computer Engineering, University of Florida, Gainsville, FL USA; 3 Department of Civil and Environmental Engineering, Tokyo Institute of Technology, Tokyo, Japan; 4 Department of Construction Management, University of Houston, Houston, TX USA

Correction to: *Communications Engineering* 10.1038/s44172-022-00032-5, published online 03 November 2022.

In this article the affiliation details for Zhijie (Sasha) Dong were incorrectly given as ‘Department of Information and Logistics Technology, University of Houston, Sugar Land, TX, USA’ but should have been ‘Department of Construction Management, University of Houston, Houston, TX, USA’. The original article has been corrected.

